# Intractable Vomiting and Hiccups: An Atypical Presentation of Neuromyelitis Optica

**DOI:** 10.7759/cureus.6245

**Published:** 2019-11-27

**Authors:** M. Mukhyaprana Prabhu, Upasana Agrawal

**Affiliations:** 1 Internal Medicine, Kasturba Medical College, Manipal, IND

**Keywords:** aquaporin-4 antibodies(nmo antibodies), neuromyelitis optica, neuromyelitis optica spectrum disorders, inflammatory disorder, intractable vomiting, hiccups

## Abstract

Neuromyelitis optica is an inflammatory disorder of the central nervous system. It involves the immune-mediated demyelination of predominantly the optic nerves and the spinal cord, which can lead to optic neuritis and transverse myelitis, respectively. Patients usually present with symptoms related to the eyes or the spinal cord, like loss of vision, pain in the eyes, visual field defects or numbness and weakness of limbs. Vomiting and hiccups are common cases encountered in medicine clinics and can sometimes be an atypical presentation of this disorder. Here we present a case of a 33-year-old female who initially presented to our tertiary care centre with repeated episodes of bilious vomiting and intractable hiccups for 10 days. After multiple investigations over a couple of days, the patient was found to be positive for anti-NMO antibodies and displayed neuro-radiological findings on MRI brain and spine, which finally led to the diagnosis of neuromyelitis optica spectrum disorder (NMOSD). Through this case we highlight the importance of suspecting NMO in a patient with complaints of intractable vomiting and hiccups, so that early intervention and treatment can prevent further disabling complications of the disease.

## Introduction

Neuromyelitis optica (previously called Devic’s disease) constitutes one of the inflammatory disorders of the central nervous system. It most commonly involves the demyelination of the optic nerves and the spinal cord, which is primarily immune-mediated. Neuromyelitis optica spectrum disorder (NMOSD) is a variant of NMO, which is usually associated with the presence of serum NMO-IgG antibody that selectively binds aquaporin-4 [1]. Optic neuritis and transverse myelitis are usually the most common presenting symptoms of the disease [2]. Since intractable vomiting and hiccups are common cases encountered in medicine clinics, they may not always raise a suspicion for neuromyelitis optica. Here we present a case of a 33-year-old female who initially presented to the tertiary care centre with repeated vomiting and intractable hiccups. A few days later after admission and further investigations, she was finally diagnosed with NMOSD.

## Case presentation

A 33-year-old female patient with nil premorbid conditions came to our tertiary care centre with complaints of vomiting for the past 10 days, which was followed by intractable hiccups for eight days. The patient was having 10-20 episodes of vomiting per day, which was bilious and small in quantity. It was not associated with fever, pain abdomen or loose stools. Hiccups for the past eight days were continuous and present throughout the day. The patient also had slurring of speech for five days which was sudden in onset and progressive. There was no history of double vision, blurring of vision, cough while swallowing food or nasal regurgitation of food. No history of weakness of limbs, loss of sensation, deviation of angle of mouth or eye closure. Bowel and bladder habits were normal. The patient had no history of similar episodes in the past. On examination at time of presentation, her vitals were stable. On central nervous system (CNS) examination, her higher mental functions were normal. Glasgow Coma Scale was E4V5M6 = 15/15. Her speech was slurred and had a guttural quality to it. Signs of cerebellar or meningeal disease were absent. Cranial nerve examination revealed deviation of palate to the right and absence of gag reflex indicating cranial nerve 9 palsy. Tongue was midline with normal movements, but tongue fasciculations were present indicating cranial nerve 10 palsy. Motor examination displayed normal tone and power in both upper and lower limbs. Reflexes were brisk bilaterally (+++) and a down-going plantar response was seen. Sensory examination showed no sensory deficits. Examination of the other systems was unremarkable.

**Figure 1:**
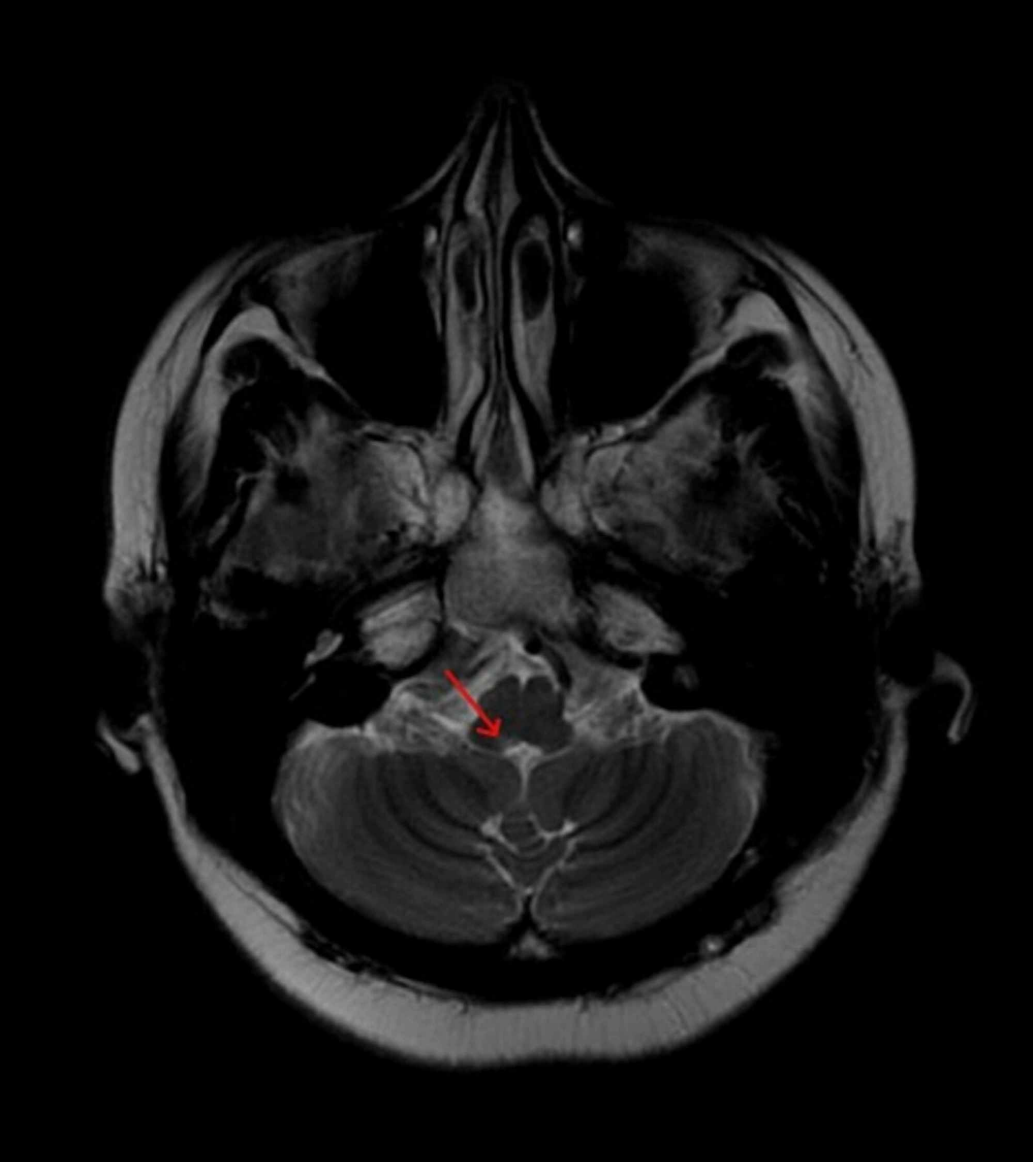
MRI brain showing a focus of active demyelination in the region of the dorsal medulla.

**Figure 2:**
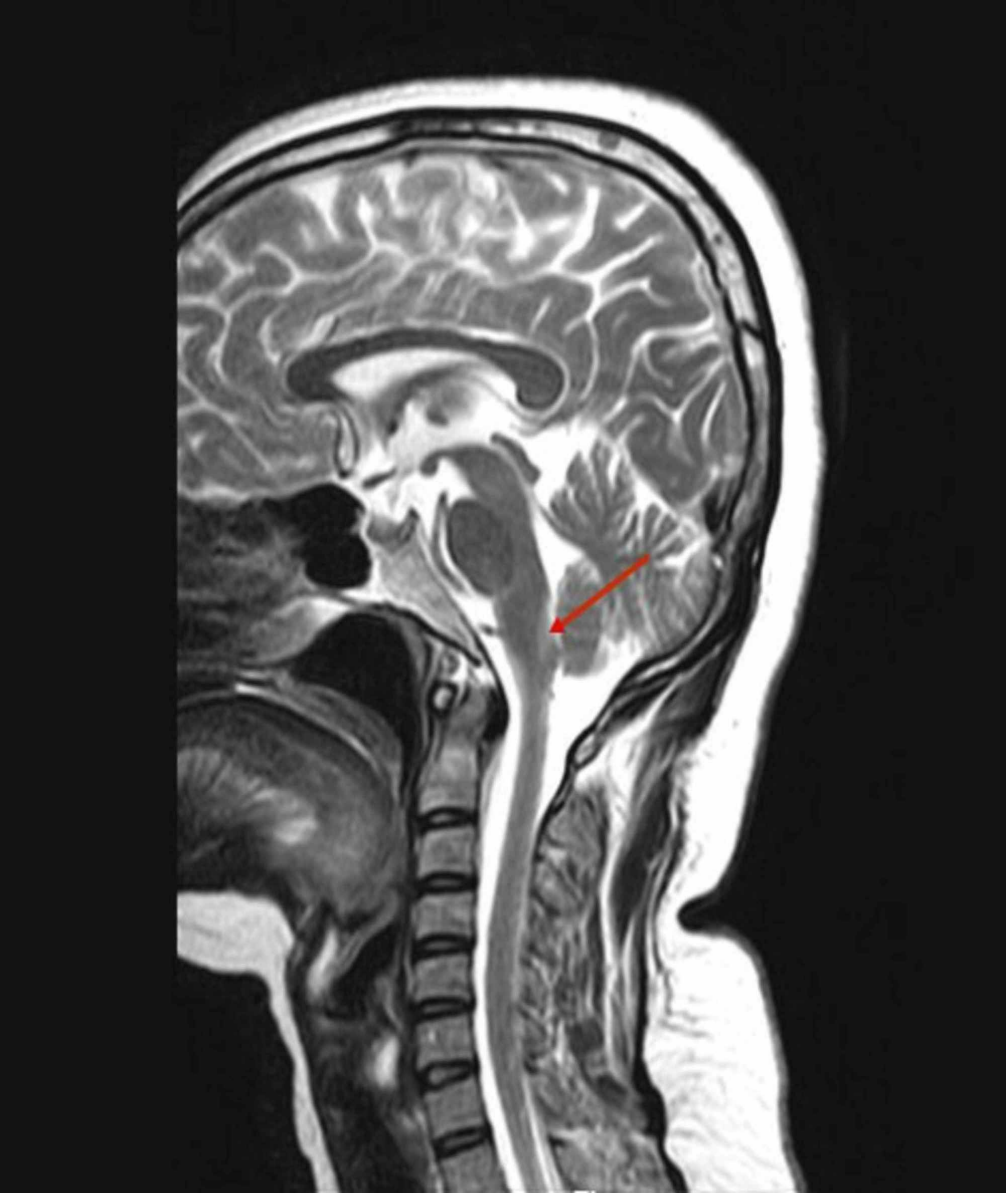
MRI spine showing an area of active demyelination in the periaqueductal region of dorsal medulla.

Investigations revealed normal blood counts and serum electrolytes. Cerebrospinal fluid (CSF) analysis was normal. Visual evoked potential (VEP) and brainstem-evoked response audiometry (BERA) tests showed no abnormalities. MRI brain showed a hyper intensity in the dorsal medulla (more on the right side) suggestive of active demyelination (Figure 1). MRI spine showed demyelination in the periaqueductal region of the dorsal medulla (Figure 2). Rest of the spine was normal. A few days later, her anti-aquaporin antibodies were reported to be strongly positive, which finally led to the diagnosis of NMOSD.

The patient was treated with intravenous methylprednisolone (IVMP) (500 mg, once a day) for five days. A decrease in symptoms was noted. At the time of discharge, the patient was stable and was advised to continue oral steroids for three weeks. Follow-up was advised for discussing tapering off of steroids and management with alternate immunosuppressive drugs.

In the follow-up visits, the patient reported a decrease in her neurological symptoms, but she started complaining of bilateral hip pain, which after an MRI of the pelvis and hip was diagnosed to be due to bilateral avascular necrosis of the femoral head. In view of avascular necrosis (AVN) of the femoral head, steroids were stopped and the patient was started on an immunosuppressant (azathioprine) and also advised physiotherapy. In further follow-up visits, she reported feeling better with a decrease in her symptoms.

## Discussion

Neuromyelitis optica (NMO) and neuromyelitis optica spectrum disorders (NMOSD) are a group of inflammatory disorders of the CNS characterized by immune-mediated demyelination and axonal damage, with primary target sites being the optic nerves and the spinal cord [1]. The usual presentation often involves sudden onset, severe episodes of optic neuritis and longitudinally extensive transverse myelitis [3]. The disease is most commonly associated with the presence of serum NMO-IgG antibodies that selectively bind aquaporin-4 (AQP4) and are highly disease specific [1].

Our case closely relates to a study done in China where 12 cases of NMO were reported and intractable vomiting and hiccups were the only initial manifestations of the first attack. Attacks involving spinal cord and optic nerves developed later [4]. Until sometime back, NMO was considered to be a clinical variant of multiple sclerosis (MS) [5]. Immunologically, NMO can be distinguished from multiple sclerosis by positive serum autoantibody NMO-IgG, which targets aquaporin-4, a transmembrane protein found on the cell membranes of cells in the CNS and spinal cord [6,7]. Serum aquaporin-4 test helps in identifying a larger variety of clinical and radiological characteristics that are related to NMO [8]. Diagnostic criteria were established in 2006 for NMOSD, which is a group of disorders clinically related to NMO. The diagnostic criteria for NMOSD require the patient to have an NMO-IgG seropositive status, along with a limited form of NMO, or the presence of symptoms such as intractable nausea, vomiting or hiccups [8]. In a study done in China, out of the 12 patients of NMO, it was observed that 83% of the patients were positive for serum aquaporin-4 antibodies (AQP4-Ab) and 60% to 70% patients had medullary lesions on MRI brain, which specifically involved the dorsomedial and area postrema regions [4]. Another study from Pakistan consisted of an 18-year-old female whose presenting complaint was of intractable vomiting and a few days later she was given the diagnosis of neuromyelitis optica [9]. However, a study from Mayo Clinic concluded that intractable vomiting is the initial presenting complaint in only 12% of the patients with neuromyelitis optica [10]. A striking observation has been made which suggests that the lesions caused by NMOSD in area postrema are associated with loss of AQP4 immunoreactivity and with inflammation, but they are different from lesions in the optic nerve and spinal cord because they lack demyelination and necrosis. This difference could be the explanation for the almost complete remission of symptoms from these lesions [11].

A study by Popescu et al. stated that the possibility of NMO presenting with nausea or vomiting is almost 16 times more with lesions in area postrema [12]. In fact an entity called area postrema syndrome (APS) exists, which involves damage to the area postrema, the bulbar region and the emetic reflex center, causing the patient to have hiccups, nausea, and/or uncontrollable vomiting. This region is particularly rich in aquaporin-4, which is the primary site of attack by anti-AQP4 antibodies responsible for NMOSDs. Therefore, APS is considered to be one of the most specific clinical manifestations of NMOSDs [13]. Since most APS attacks precede the inflammatory attack on the optic nerves or spinal cord, this makes it an important warning sign.

Acute attacks of NMOSD are usually treated with 1,000 mg of IVMP for three to five days [14]. However in our case, 500 mg of IVMP was effective in controlling the symptoms. For prevention of further attacks, it is recommended to start oral steroids for the initial few months, along with azathioprine or mycophenolate mofetil until these drugs reach their full efficacy [14]. But one disadvantage of systemic corticosteroid therapy is the risk of development of necrosis of bone [15]. This was witnessed in our patient, who developed avascular necrosis of bilateral femoral heads within a few weeks of the start of systemic steroid therapy.

## Conclusions

Since vomiting and nausea are usually one of the common and non-specific presenting symptoms to the hospital and can usually be traced to gastrointestinal causes, physicians initially do not associate these symptoms with a neurological cause. This can lead to a delayed diagnosis of disorders such as neuromyelitis optica (NMO). Our case report highlights the importance of early diagnosis and treatment of NMOSD, which can not only help reduce the severity of the attacks, but can also help in preventing attacks of optic neuritis or transverse myelitis which can prove to be disabling for the patient if not controlled early.
